# Selective Regulation of NR2B by Protein Phosphatase-1 for the Control of the NMDA Receptor in Neuroprotection

**DOI:** 10.1371/journal.pone.0034047

**Published:** 2012-03-30

**Authors:** Mélissa Farinelli, Fabrice D. Heitz, Benjamin F. Grewe, Shiva K. Tyagarajan, Fritjof Helmchen, Isabelle M. Mansuy

**Affiliations:** 1 Brain Research Institute, University of Zürich, Zürich, Switzerland; 2 Department of Biology, Swiss Federal Institute of Technology, Zürich, Zürich, Switzerland; 3 Institute of Pharmacology and Toxicology, University of Zürich, Zürich, Switzerland; The University of Sydney, Australia

## Abstract

An imbalance between pro-survival and pro-death pathways in brain cells can lead to neuronal cell death and neurodegeneration. While such imbalance is known to be associated with alterations in glutamatergic and Ca^2+^ signaling, the underlying mechanisms remain undefined. We identified the protein Ser/Thr phosphatase protein phosphatase-1 (PP1), an enzyme associated with glutamate receptors, as a key trigger of survival pathways that can prevent neuronal death and neurodegeneration in the adult hippocampus. We show that PP1α overexpression in hippocampal neurons limits NMDA receptor overactivation and Ca^2+^ overload during an excitotoxic event, while PP1 inhibition favors Ca^2+^ overload and cell death. The protective effect of PP1 is associated with a selective dephosphorylation on a residue phosphorylated by CaMKIIα on the NMDA receptor subunit NR2B, which promotes pro-survival pathways and associated transcriptional programs. These results reveal a novel contributor to the mechanisms of neuroprotection and underscore the importance of PP1-dependent dephosphorylation in these mechanisms. They provide a new target for the development of potential therapeutic treatment of neurodegeneration.

## Introduction

N-methyl-D-aspartate receptors (NMDARs) are essential receptors for excitatory neurotransmission and synaptic plasticity in the nervous system. The activity of these receptors is tightly controlled by pre-synaptic glutamate release and intracellular protein kinases and phosphatases through post-translational modifications. Alterations in NMDAR functions and in the balance between downstream kinases and phosphatases can occur in pathological conditions, and lead to neuronal excitotoxicity. Excitotoxicity is a detrimental cellular process that results from excessive glutamate release, subsequent over-activation of NMDARs and intracellular calcium (Ca^2+^) overload. While high Ca^2+^ influx through NMDARs is generally deleterious and activates signaling pathways inducing cell death, it can however also be beneficial and stimulate cell survival pathways leading to neuroprotection [1], [2], [3]. The mechanisms that control the recruitment of cell death or cell survival pathways upon activation of NMDARs are thought to depend in part, on the Ca^2+^ concentration and its route of entry, but mostly on the subunit composition and localization of the NMDARs that it activates [4], [5]. Several pieces of evidence have suggested that heteromeric NR1/NR2B receptors are initial triggers of cell death pathways, while NR1/NR2A receptors rather lead to cell survival [6]. First, in mature cortical cultures and in rat *in vivo*, the activation of NR2B-containing NMDARs results in excitotoxicity, while the activation of NR2A-containing NMDARs promotes neuroprotection [6]. Second, NR2B-containing NMDARs are thought to be localized preferentially at extrasynaptic sites while NR2A-containing NMDARs are synaptic [7], [8], and activation of extrasynaptic NMDARs and associated downstream signaling cascades correlates with a pro-death transcriptional response while activation of synaptic NMDARs lead to pro-survival transcriptional response [9]. Third, neurotoxicity induced by glutamate release from astrocytes involves preferentially extrasynaptic NR2B-containing NMDARs [10], [11]. Finally, glutamate sensitivity in neurons increases proportionally to the level of NR1/NR2B expression [12] as NR2B-containing NMDARs have a higher affinity for glutamate, slower deactivation kinetics, and reduced Ca^2+^-dependent desensitization when compared to NR2A-containing receptors [13]. Despite the multiple evidence supporting a role for NR2B-containing NMDARs in excitotoxicity however, the major regulators of these receptors have remained undefined.

Protein phosphorylation is a post-translational modification that constitutes one of the major modes of regulation of the NMDAR. Phosphorylation controls the functional properties of NMDARs and the surface expression of NMDAR subunits (Lee 2006, Salter 2004, Prybylowski 2004). While several protein kinases are known to phosphorylate the NMDAR i.e. death-associated protein kinase 1 (DAPK1) [14], Ca^2+^/calmodulin (CaM)-dependent protein kinase II alpha (CaMKIIα) [15], PKA [16], PKC [17] and Src family kinases [18], it is not entirely clear which protein phosphatase(s) can induce dephosphorylation. This is an important limitation because dephosphorylation is as essential as phosphorylation for the control of the receptor's activity. One of the potential protein phosphatases that may be involved is protein phosphatase 1 (PP1). It has several features that make it a likely candidate for regulating the NMDAR. PP1 is highly abundant in neurons, and following NMDAR activation, it is recruited to the receptor and associates with it by binding to specific targeting partners. There, it decreases NMDAR activity and synaptic strength by reducing the receptors open probability [19], [20]. Further, when activated in hippocampal neurons, for instance after induction of synaptic plasticity, PP1 can exert neuroprotective functions, while in contrast, its inhibition exacerbates the detrimental effect of excitotoxicity [21].

This study examined the contribution of PP1 to the regulation of the NMDAR in CA1 hippocampal neurons, and its implication in neuroprotective pathways. We used oxygen-glucose deprivation (OGD) in organotypic hippocampal cultures to induce NMDAR-dependent neuronal excitotoxicity [22]. It involves mechanisms such as membrane depolarization with calcium entry into the cell and reactive oxygen species (ROS) production. We demonstrate that PP1α can regulate NR2B-containing NMDARs by dephosphorylating a specific residue on NR2B, and that this leads to a reduction in Ca^2+^ overload and an attenuation of excitotoxic damage. The data shows that PP1α promotes key signaling cascades downstream of the NMDAR through altered phosphorylation, and can thereby initiate pro-survival programs.

## Materials and Methods

### Vector construction, virus production and titration

The lentiviral vector pLVPRT-tTRKRAB [23] was used as a backbone to generate pLVPRT-tTRKRAB-PP1α and pLVPRT-tTRKRAB-PP1α-EGFP for inducible PP1α expression in neurons. Full-length coding sequence of human (mutated rabbit) PP1α alone or fused with EGFP (PP1α or PP1α-EGFP) from pEGFP-C1-PP1α [24] was subcloned between the MluI and SmaI sites of pLVPRT-tTRKRAB. The constructs were sequenced before virus production. Recombinant lentiviruses were produced as previously described [23] using pLVPRT-tTRKRAB-PP1α (PP1α virus), pLVPRT-tTRKRAB-PP1α-EGFP (PP1α-EGFP virus), a native pLVPRT-tTRKRAB vector (control virus), a packaging encoding vector psPAX2 and the envelope encoding vector pMD2G. Viral particles were titrated using the HIV-1 p24 ELISA test (PerkinElmer), according to the manufacturer's protocol.

### Slice culture and viral infection

Organotypic hippocampal slices from 5–7 day old postnatal wild-type C57/BL6 or I-1 transgenic mice were prepared and cultured for 2 weeks using the roller-tube technique [25]. Slices prepared from wild-type C57/BL6 mice were transferred to an empty electrophysiological recording chamber at room temperature and the CA1 pyramidal cell layer was injected with lentiviral preparations (∼2.10^8^ TU/ml, 5–10 injection sites, total volume of 2 µl). Artificial cerebrospinal fluid (aCSF): (in mM) 119 NaCl, 2.5 KCl, 26 NaHCO_3_, 1.3 NaH_2_PO4, 1.3 MgCl_2_, 2.5 CaCl_2_, and 11 D-glucose, pH 7.4, saturated with 95% O_2_, 5% CO_2_) was used for control injections. Slices were returned to roller tubes after injection, and cultured for 1–2 weeks in medium supplemented with penicillin/streptomycin (1∶500; Sigma). One day before analysis, culture medium was supplemented with 1 µg/ml doxycycline hyclate (Sigma) to induce PP1α-EGFP, PP1α or I-1 transgene expression. All animal experiments have been approved by the federal veterinary office (FVO).

### Induction of oxygen-glucose deprivation (OGD)

Slices were taken out of the sealed test tubes and washed once in normal aCSF (see slice culture and viral infection) before OGD was induced. For this, normal aCSF solution was replaced with an aCSF solution deprived of oxygen (saturated with 95% N2, 5% CO2) and glucose (replaced with 11 mM sucrose) and slices were put back in the incubator for 4 min. Initial conditions were restored after the 4-min OGD.

### Quantitative real-time RT-PCR

For each sample, total RNA from three hippocampal slices was extracted using the NucleoSpin Kit II (Macherey-Nagel), purified with Promega's RQ1 DNase and reverse-transcribed using the SuperScript First-Strand Synthesis System for RT-PCR II (Invitrogen). Quantitative PCR was performed using Taqman probes (Applied Biosystems) and an Applied Biosystem 7500 Thermal Cycler. Each sample was analyzed in triplicate and equal amount of cDNA was plated. Values were chosen in the linear range of amplification and comparative Ct method was used to assess difference in gene expression between samples [26]. β-actin was used as internal control.

### Immunohistochemistry

Slices were fixed overnight at 4°C in 4% paraformaldehyde (Sigma), 0.1 M phosphate buffer (PB), pH 7.4. Free-floating sections were washed in 0.1 m PB (3×1 h), blocked and permeabilized in 0.1 M PB, 0.4% Triton X-100 (Sigma) and 10% heat-inactivated horse serum (HS; Sigma) for 24 h at 4°C. Slices were incubated with primary rabbit anti-EGFP (Synaptic Systems) and anti-NeuN (Chemicon) antibodies (1∶1000) for 72 h at 4°C in 0.1 M PB, 0.4% Triton X-100 and 10% HS. Cultures were washed in 0.1 M PB 0.4% Triton X-100 (3×1 h) and incubated overnight at 4°C with goat anti-rabbit IgG-FITC and donkey anti-mouse IgG-TRITC fluorescence-conjugated secondary antibodies (1∶1000; Jackson ImmunoResearch). After washing in 0.1 M PB, cultures were mounted using Moviol (Molecular Probes) and stored in the dark at 4°C. Fluorescent images at low magnification were acquired with a CoolSNAPK4 digital camera (Roper Scientific) mounted on an Axiophot microscope (Zeiss) and analysed using MCID Elite 7.0 software (MCID). High magnification images were taken with a Zeiss LSM 410 confocal laser-scanning microscope using excitation wavelengths of 543 nm (TRITC) and 488 nm (FITC), and images were averaged to improve signal-to-noise ratio.

### Whole-cell recording

Hippocampal slices injected with aCSF, PP1α, PP1α-EGFP or control virus treated with doxycycline were recorded in whole-cell configuration. After 15 min of baseline, slices were exposed to 4 min OGD (hypoxic/aglycemic conditions) by switching the perfusion from normal aCSF (see slice culture and viral infection) to an aCSF solution deprived of oxygen (saturated with 95% N2, 5% CO2) and glucose (replaced with 11 mM sucrose). After the 4-min OGD, perfusion with normal aCSF was reinstalled. Recordings were obtained from CA1 pyramidal neurons at −50 mV. Patch pipettes (4–8 MΩ) were filled with (in mM) 140 K-gluconate, 10 NaCl, 1 MgCl_2_, 10 HEPES, and 1 EGTA (pH 7.2–7.4). Series resistance, typically between 10 and 20 MΩ, was regularly monitored and if a change of more than 10% occurred, cells were excluded. NMDA (100 µM, Sigma) was pressure ejected (1 bar, 200 ms) at 40 s intervals from a pipette positioned ∼100 µm from the soma of recorded cells. To isolate the evoked NMDA receptor-mediated currents, the AMPA/kainate receptor antagonist NBQX 10 µM, the GABA_A_ antagonist picrotoxin 100 µM (Sigma) and the voltage-gated Na^+^ channel blocker tetrodotoxin 1 µM were added to the bath solution. Specificity of NMDA receptor currents was verified at the end of recordings by application of a modified aCSF without Mg^2+^ and containing the non-competitive NMDA receptor antagonist MK-801 (10 µM). Currents were filtered at 1–2 kHz, stored, and analyzed off-line. Individual responses to NMDA were measured from the holding current to the peak and the OGD effect was expressed relative to the pre-conditioning baseline (mean response over 5 min prior to OGD and normalized to 100%). Ifenprodil (3 µM; Tocris Bioscience), NVP-AAM077 (0.1 µM; kindly provided by Y. Auberson, Novartis, Basel, Switzerland) or KN-93 (5 µM; Calbiochem) were added to the perfusion 15 min before recording of the pre-conditioning baseline and maintained through recording. After 5 min of baseline, slices were exposed to 4-min OGD (as above). Recordings were made using an Axopatch 200B amplifier (Axon Instruments), monitored on-line and analyzed off-line using pCLAMP. Data were pooled across slices and expressed as mean ± SEM.

### Ca^2+^ imaging

CA1 pyramidal cells were filled with the Ca^2+^ indicator Oregon Green 488 BAPTA-1 (20 µM; Invitrogen) via whole-cell recording patch pipettes (4–8 MΩ; series resistance 10–20 MΩ) as previously described [27]. Cells were clamped at −50 mV as described in the whole-cell recording section. The indicator was added to the intracellular solution and excited at 488 nm using a Polychrome I monochromator from TILL Photonics (Planegg). Fluorescence images were collected with a cooled CCD camera (Princeton Instruments) after passing through a FITC emission filter set, and stored using a software custom-written in the LABVIEW environment (National Instruments). Time series of 50 images were collected at 15 sec intervals (exposure time 100 msec) and analyzed using ImageJ software. The time course of relative fluorescence changes (ΔF/F) was analyzed after background subtraction in regions of interest over the soma as the percentage ratio (F-F_0_)/F_0_, where F_0_ is the average fluorescence during the baseline period.

### Co-expression of NR2B and TN-XXL calcium indicator

The NR2B mutated construct (NR2B S1303A) was produced by PCR-based mutagenesis (QuikChange Site-Directed Mutagenesis Kit, Stratagene) using full-length NR2B within pRK7-NR2B. Both pRK7-NR2B (NR2B S1303 WT) and pRK7-NR2B S1303A (NR2B S1303A) constructs were verified by sequencing. To achieve NR2B expression and subsequently quantify [Ca^2+^]_i_ load in CA1 pyramidal neurons, organotypic hippocampal cultures (DIV14-17) were co-transfected with one of the NR2B constructs (NR2B S1303 WT and S1303A) and the TN-XXL construct encoding a calcium indicator [28], using Gene Gun (BioRad) biolistic transfection. Slices transfected with TN-XXL construct alone were used as control. Successfully transfected cells were subjected to OGD and analyzed by Ca^2+^ imaging.

### Propidium iodide labeling

Slice cultures were washed 3 times in culture tubes and incubated for 4 min with either ACSF (control) or the OGD solution in an incubator at 35±1°C. Cultures were washed 3 times with ACSF and returned to culture medium containing 2.5 µg/ml propidium iodide (Sigma) and penicillin/streptomycin (1∶500; Sigma). After 48 h, cultures were photographed with constant settings using a CoolSNAPK4 camera (Roper Scientific) mounted on an Axiophot microscope (excitation 546 nm, emission >590 nm, Zeiss). Images were captured using the same acquisition parameters and visualized with MCID Elite 7.0 software (MCID), blind to the treatment group. Images were analyzed using ImageJ software (NIH) by drawing and positioning a circle of 6000 pixels (diameter of about 0.1 mm) over the brightest region of CA1 pyramidal layers. The normalized change in average intensity was calculated by subtracting background fluorescence.

### Western blotting

Hippocampal slices were pooled by three and homogenized using a 26 G needle syringe in 10 mM HEPES, 1 mM MgCl2, 5 mM EDTA, 0.2% (v/v) Triton X-100 (Sigma), 10% (v/v) glycerol (Sigma), protease inhibitor cocktail (Sigma), phosphatase inhibitor cocktails 1 and 2 (Sigma), 250 µM PMSF (Sigma), and 15 mM β-mercaptoethanol (Sigma). 20 to 30 µg total protein were resolved on 8–10% SDS-PAGE and transferred onto a nitrocellulose membrane (BioRad). Membranes were blocked (Rockland IR blocking buffer, Rockland), and incubated in primary antibodies (1∶1000): rabbit anti-mouse phospho-NMDAR2B Ser 1303 (Upstate), rabbit anti-mouse phospho-NMDAR2B Tyr1472 (Sigma), mouse anti-mouse NMDAR2B (Chemicon), rabbit anti-mouse phospho-CaMKIIα/β Thr286/287 (Upstate), rabbit anti-mouse CaMKIIα (Sigma), mouse anti-mouse phospho-CREB Ser133 (Cell Signaling Technology), rabbit anti-mouse CREB (Upstate), and mouse anti-mouse β-actin (Sigma). Membranes were incubated in secondary antibodies (1∶10000): goat anti-rabbit and anti-mouse IRDye 680, goat anti-rabbit and anti-mouse IRDye 800 (Li-Cor Biosciences). Band intensity was determined and quantified using an Odyssey IR scanner (Li-Cor Biosciences). The phospho-protein signal was normalized to the corresponding protein signal, and β-actin was used as a loading control. Values from PP1α-expressing and control cultures subjected to OGD were normalized to values from control cultures where OGD was not induced.

### Co-immunoprecipitation

Protein samples from whole hippocampus lysates were prepared by homogenization in 500 µl sterile-filtered 50 mM Tris, 120 mM NaCl, 0.5% NP-40 containing proteinase and phosphatase inhibitors (1∶100; Sigma), followed by centrifugation for 15 min at 14,000 rpm at 4°C and collection of the supernatant. After 60 to 90 min incubation with 1–2 µg of the corresponding antibody, 20 µl of BSA-precleared pansorbin-protein A beads (Calbiochem) were added for 45 min at 4°C. The immune complexes were spun at 8000 rpm for 3 min, washed in high-salt homogenization buffer (containing 50 mM Tris, 500 mM NaCl and 1% NP-40) and regular homogenization buffer. Samples were loaded on a 8% SDS gel and analyzed (see Western blotting). IP antibodies used were rabbit polyclonal anti-PP1α (Chemicon) and rabbit anti-IgG (Sigma) as control, and blotting antibodies were rabbit polyclonal anti-NR2B (Millipore) and mouse monoclonal anti-NR1 C-terminal (Upstate).

### NR2B phosphorylation assay

The GST-NR2B fusion peptide including the NR2B C-terminal region tail (residues 1221–1501) was expressed in *E.Coli* from the pGEX-NR2B vector [29] and purified according to Amersham Biosciences protocol. *In vitro* NR2B phosphorylation/dephosphorylation was conducted using purified recombinant CaMKII and PP1 enzymes (New England Biolabs), according to supplied protocols. Products were loaded on a 8–10% SDS gel and phosphorylation was analyzed (see Western blotting).

### Statistical analysis

Data are presented as mean normalized to baseline or control ± SEM. Paired Student's t-tests were used to compare non-normalized data. Statistical significance was set at p≤0.05(*), p≤0.01(**) and p≤0.001(***).

## Results

### PP1α overexpression in CA1 hippocampal neurons

**Figure 1:**
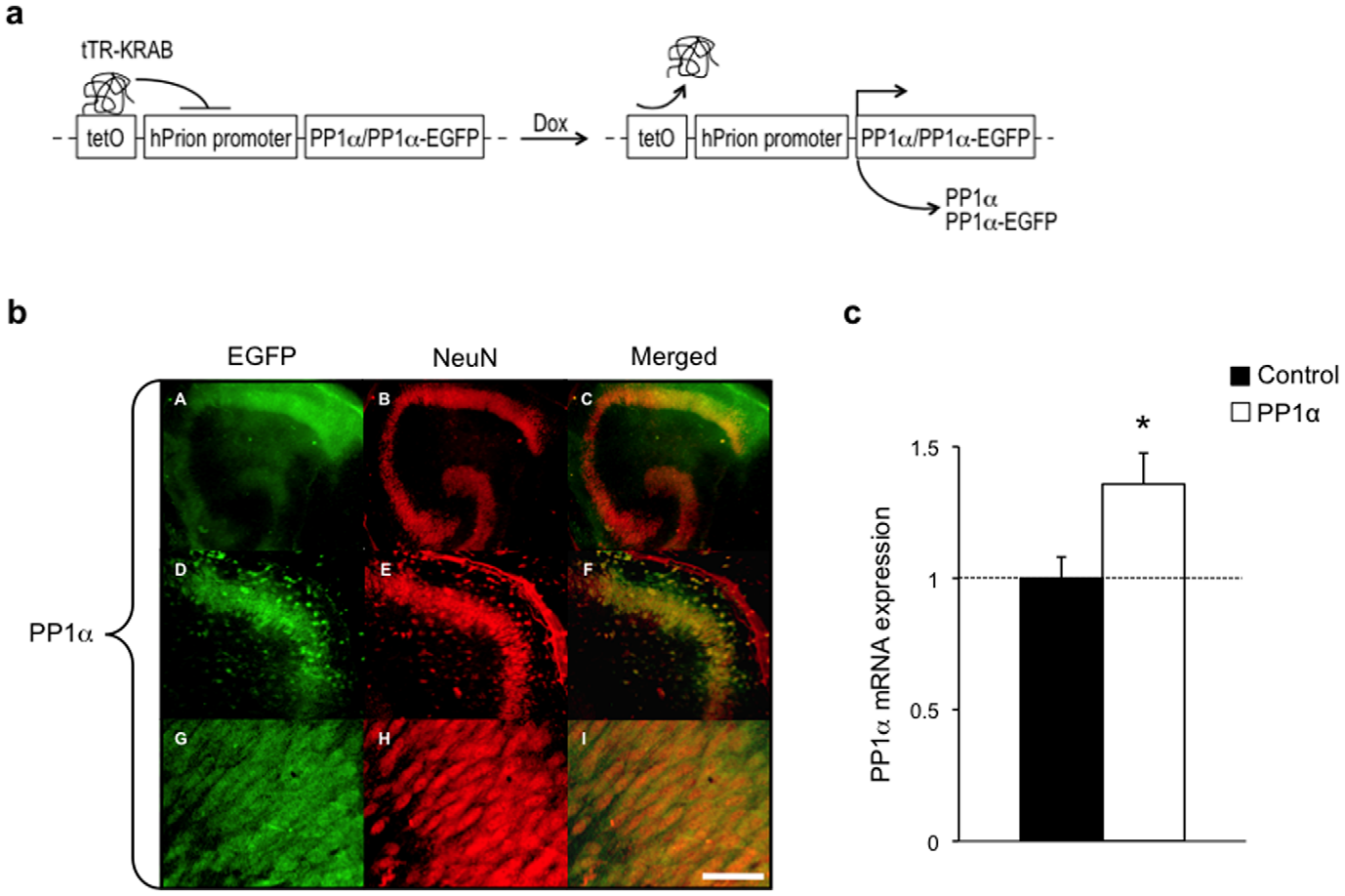
Inducible and neuron-specific PP1α expression in hippocampus. (a) Schematic representation of the genetic system used to achieve doxycycline-dependent and neuron-specific PP1α or PP1α-EGFP expression in mouse organotypic hippocampal slices. Upon doxycycline treatment, binding of the tetracycline repressor and Krüppel-associated box fusion protein (tTR-KRAB) to the tetracycline response element (tetO) is blocked, which prevents the epigenetic silencing of the human prion (hPrion) promoter and allows PP1α or PP1α-EGFP expression. (b) EGFP (green) and NeuN (red) co-immunostaining in CA1 area showing neuron-specific PP1α-EGFP expression. Scale bar, 400 µm in A–C, 200 µm in D–F, and 25 µm in G–I. (c) Real-time quantitative RT-PCR showing increased PP1α mRNA expression in slices injected with PP1α (n = 14) compared to control slices (n = 8). Data is expressed as relative quantification. *p<0.05. Error bars represent mean SEM.

To address the importance of PP1 in NMDAR-dependent excitotoxicity in the adult hippocampus, we conditionally expressed PP1α in CA1 hippocampal neurons using a lentivirus approach. We chose CA1 neurons because they are highly vulnerable to excitotoxicity [30]. We generated vectors expressing PP1α alone (PP1α) or PP1α fused to enhanced green fluorescent protein (PP1α-EGFP) under the control of the neuron-specific human prion (hPrion) promoter (**Figure 1a**). These vectors were used to overexpress PP1 in organotypic hippocampal slices. In the slices injected with the PP1α-EGFP vector, EGFP/NeuN co-staining confirmed the neuronal specificity of PP1α expression, and showed that expression is homogenous and mainly distributed across CA1 neurons but is also present in CA3 and dentate gyrus neurons (**Figure 1b**). Quantitative real-time RT-PCR showed that the PP1α vector increased PP1α mRNA expression in the hippocampus by 36±11% (**Figure 1c**). PP1α protein shows a somato-dendritic distribution with enrichment in dendritic spines (**Figure S1a and Supporting Methods S1**), suggesting its presence at glutamatergic synapses. In contrast, the other major catalytic isoform PP1γ is enriched in the nucleus (**Figure S1b and Supporting Methods S1**).

### PP1α overexpression reduces [Ca^2+^]_i_ overload and prevents cell death during excitotoxicity

**Figure 2:**
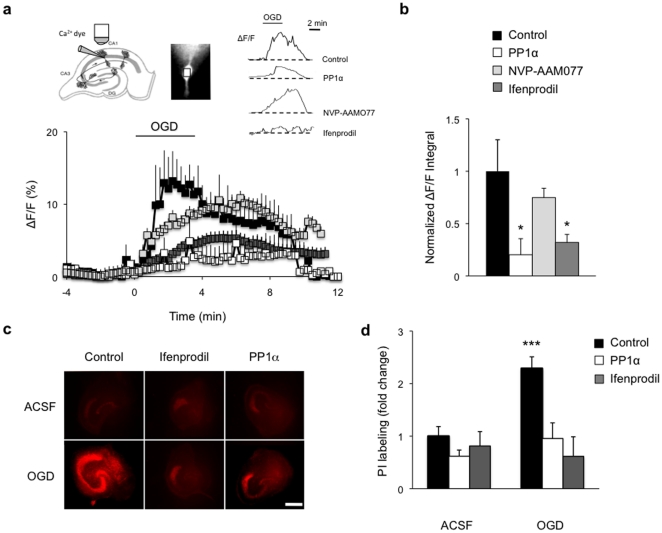
Blockade of Ca^2+^ rise and delayed cell death induced by OGD by ifenprodil and PP1α in CA1 pyramidal cells. (**a**) OGD-induced increase in ΔF/F (% relative to basal level) in control and NVP-AAM077-treated slices (control, n = 7; NVP-AAM077, n = 7). This increase is prevented by ifenprodil perfusion (n = 8) or by PP1α expression (n = 7). Schematic representation of a hippocampal slice showing the patch-clamp electrode filled with OGB-1 (left inset). Image of a CA1 pyramidal neuron loaded with OGB-1 with outlined region of interest (cell soma) used to calculate fluorescence (middle inset). Relative fluorescence (ΔF/F) traces of individually recorded CA1 pyramidal neurons in control slices, PP1α expressing slices, and slices treated with NVP-AAM077 or ifenprodil subjected to OGD (right inset). (**b**) Quantitative histogram illustrating the area under the curve used as a measure of intracellular Ca^2+^ overload during 4 min OGD, given by the calculation of ΔF/F integral normalized to control. *p<0.05. (**c**) Propidium iodide labeling of hippocampal slices showing massive cell death in the CA1 pyramidal cell layer (red staining) 48 h after OGD (control OGD, n = 7). Ifenprodil treatment (n = 6) and PP1α expression (n = 5) significantly reduced cell death as shown by an almost absent PI labeling after OGD. No cell death was observed in slices maintained in ACSF (control ACSF, n = 8; ifenprodil ACSF, n = 8; PP1α ACSF, n = 7). Scale bar: 400 µm. (**d**) Quantitative histogram with normalized labeling intensity. ***p<0.001.

A critical step in the mechanisms of excitotoxicity is the over-activation of NMDARs and the resulting overload in intracellular Ca^2+^ ([Ca^2+^]_i_) [31]. Because the functions and properties of the NMDAR depend on its subunit composition [4], [5], it is important to first determine which receptor subtype contributes to the increased Ca^2+^ influx following excitotoxic insult. We used oxygen/glucose deprivation (OGD) as paradigm to induce excitotoxicity in hippocampal neurons and examined the Ca^2+^ influx and NMDAR subunit composition by Ca^2+^ imaging and electrophysiological recording. Post-synaptic Ca^2+^ influx was made NMDAR-dependent by blocking alpha-amino-3-hydroxy-5-methyl-4-isoxazolepropionic acid (AMPA) and kainate receptors, γ-aminobutyric acid_A_ (GABA_A_) receptors, and voltage-gated Na^+^ channels, and was visualized by loading individual CA1 pyramidal neurons with the high-affinity indicator Oregon Green 488 BAPTA-1 (OGB-1). This revealed that somatic [Ca^2+^]_i_ was rapidly and transiently increased during OGD, consistent with previous reports [32] (**Figure 2a,b**). Blockade of NR2B-containing NMDARs with ifenprodil [33] significantly attenuated this increase, and reduced the overall Ca^2+^ load by about 70% (+/−9%), suggesting the contribution of the NR2B subunit. This effect was specific to NR2B and did not involve NR2A since blockade of NR2A-containing NMDARs with 0.1 µM NVP-AAM077 [34] had no effect on [Ca^2+^]_i_. It reduced basal NMDAR-mediated responses by about 70% (data not shown), suggesting a efficient inhibition of NR2A responses with little effect on NR2B [35].

We then assessed whether PP1α contributes to the regulation of Ca^2+^ influx by NR2B-containing NMDARs, using hippocampal slices in which PP1α was either overexpressed or inhibited. We found that PP1α overexpression reduces the maximal [Ca^2+^]_i_ rise and Ca^2+^ load in hippocampal neurons, and that the reduction was comparable to that observed with ifenprodil. Conversely, PP1α inhibition, achieved by transgenic expression of an active PP1 inhibitor (I-1) [36], (**Figure S2a and Supporting Methods S1**), prolonged [Ca^2+^]_i_ rise and increased [Ca^2+^]_i_ load ( 37+/−9%) in CA1 neurons subjected to OGD (**Figure S2b and Supporting Methods S1**). These results strongly suggest that PP1α is involved in the bidirectional regulation of Ca^2+^ influx through NMDARs.

Since in excitotoxicity, Ca^2+^ overload is often associated with delayed neuronal death [31], we next examined whether ifenprodil or PP1α overexpression diminish cellular damage. To visualize cell death, we used propidium iodide (PI), a marker that rapidly penetrates cells with damaged membrane and fluoresces upon binding to DNA. A significant increase in PI staining (2.3 fold+/−0.2) was observed in pyramidal layers in area CA1–CA3 and in dentate gyrus 48 hours after OGD challenge, indicating massive neuronal death (**Figure 2c,d**). Cell death was strongly reduced in all hippocampal areas including CA1 but also CA3 and dentate gyrus by both ifenprodil and PP1α overexpression as shown by a lack of increase in PI staining in these conditions. Neuroprotection in area CA3 and dentate gyrus likely result from PP1α overexpression in CA1 region. Indeed, cell death following excitotoxic insult is known to initially affect CA1 pyramidal cells in an acute phase followed by delayed damage in area CA3 and dentate gyrus [30], [37]. The protecting effect of PP1 in area CA1 may therefore impact area CA3 and the dentate gyrus after a delay. Together, these findings strongly suggest that [Ca^2+^]_i_ overload during OGD is largely mediated by NR2B-containing NMDARs, and that such overload and the resulting cellular damage can be prevented by PP1α.

### NR2B Ser1303 phosphorylation contributes to [Ca^2+^]_i_ overload and is prevented by PP1α overexpression or CaMKII inhibition

**Figure 3:**
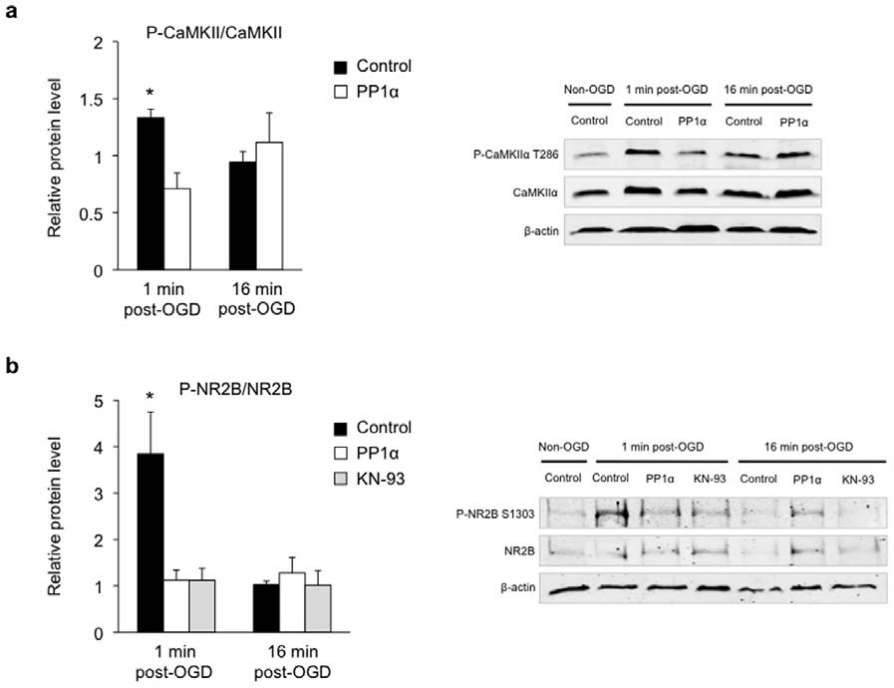
PP1α reverses CaMKIIα Thr286 phosphorylation and mediates the dephosphorylation of NR2B Ser1303 upon OGD. (**a**) Representative Western blots and corresponding quantitative analysis of CaMKIIα Thr286 phosphorylation. CaMKIIα phosphorylation significantly increases 1 min after OGD (control 1 min post-OGD, n = 8) with no significant change at 16 min (control 16 min post-OGD, n = 6) in control slices. PP1α slices do not display any change in CaMKIIα phosphorylation either 1 min (PP1α 1 min post-OGD, n = 6) or 16 min post-OGD (PP1α 16 min post-OGD, n = 7) compared to control. Phospho-protein levels were normalized to non-phosphorylated protein levels and β-actin was used as a loading control. Quantitative data for each condition were normalized to levels of non-OGD condition (control non-OGD, n = 14) from the same blot and exposure. *p<0.05. (**b**) Representative Western blots and corresponding quantitative analysis of NR2B Ser1303 phosphorylation. Increased level of phospho-NR2B in control slices 1 min after OGD (control 1 min post-OGD, n = 9), but not 16 min after OGD (control 16 min post-OGD, n = 8). PP1α expression or KN-93 treatment blocks this increase (PP1α 1 min post-OGD, n = 6; KN-93 1 min post-OGD, n = 6), but has no effect 16 min post-OGD (PP1α 16 min post-OGD, n = 8; KN-93 16 min post-OGD, n = 5). Phospho-protein levels were normalized to non-phosphorylated protein levels, and β-actin was used as a loading control. Quantitative data for each condition were normalized to levels of non-OGD condition (control non-OGD, n = 14) from the same blot and exposure. *p<0.05.

Changes in intracellular [Ca^2+^]_i_ are known to modulate Ca^2+^-dependent signaling pathways and alter the activity of Ca^2+^-activated enzymes, in particular Ca^2+^/calmodulin-dependent protein kinase II (CaMKII). CaMKII is a serine/threonine protein kinase that can autophosphorylate on several residues in the presence of Ca^2+^ [38]. Autophosphorylation on Thr286 confers autonomous Ca^2+^-independent activity to the enzyme [38], [39], [40], and promotes its association with the PSD [41]. We examined whether CaMKII Thr286 phosphorylation is modulated by OGD in our experimental conditions, and whether PP1α influences this autophosphorylation. Western blot analyses revealed that Thr286 phosphorylation was significantly increased 1 min after OGD (1.4 fold+/−0.1), a time point that corresponds to maximum [Ca^2+^]_i_ peak, then returned to baseline within 16 min in control slices (**Figure 3a**). This effect was specific to CaMKIIα phosphorylation and was not accompanied with any change in the total level of the protein. Since in the PSD, CaMKIIα can be dephosphorylated by PP1 [41], we also investigated the effect of PP1α overexpression on Thr286 phosphorylation. We observed that CaMKII Thr286 phosphorylation was significantly reduced by PP1α overexpression 1 min after OGD (**Figure 3a**). PP1α overexpression did not induce any change in CaMKII Thr286 phosphorylation.

Since the toxic [Ca^2+^]_i_ overload during OGD is largely mediated by NR2B-containing NMDARs (**Figure 2a,b**), and correlates with CaMKIIα activation by Thr286 phosphorylation, we next examined the link between CaMKIIα and NR2B-containing NMDARs. We determined whether Ser1303 on NR2B, a site phosphorylated by CaMKIIα [15], is modulated by OGD. Western blot analyses showed that Ser1303 phosphorylation was significantly increased 1 min after OGD (3.8 fold+/−0.9) and returned to baseline within 16 min in control slices, without any change in the total level of NR2B (**Figure 3b**). Similar to CaMKII phosphorylation, this increase correlated with the maximum [Ca^2+^]_i_ peak, suggesting a link between NR2B Ser1303 phosphorylation, CaMKII phosphorylation and Ca^2+^ entry. Consistently, we observed that the increase in NR2B phosphorylation was abolished by KN-93, a specific CaMKII inhibitor (**Figure 3b**). Furthermore, PP1α expression significantly reduced the level of phosphorylated NR2B Ser1303 1 min after OGD (**Figure 3b**), while no effect was observed in non-OGD conditions.

**Figure 4:**
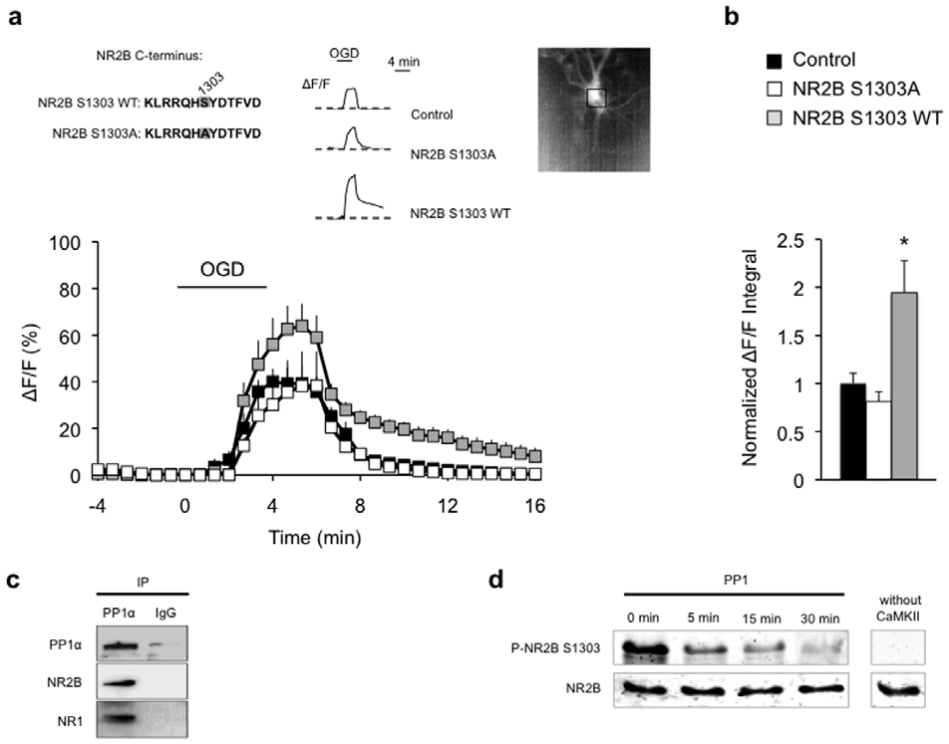
NR2B Ser1303 phosphorylation is necessary and sufficient to drive Ca^2+^ overload. (**a**) Expression of wild-type NR2B (NR2B S1303 WT, n = 6) increased OGD-induced ΔF/F (% relative to basal level), while expression of NR2B mutated on Ser1303 (NR2B S1303A, n = 5) was not changed compared to control slices (control, n = 6). C-terminus amino-acid sequence of NR2B S1303 WT and NR2B S1303A mutated version with substitution of serine with alanine on residue 1303 in shaded grey (left inset). Relative fluorescence (ΔF/F) traces of individually recorded CA1 pyramidal neurons in control, NR2B S1303A, and NR2B S1303 WT slices subjected to OGD (middle inset). Image of a CA1 pyramidal neuron expressing the calcium sensitive dye TN-XXL with outlined region of interest (cell soma) used to assess fluorescence and [Ca^2+^]_i_ (right inset). (**b**) Quantitative histogram illustrating the area under the curve used as a measure of total intracellular Ca^2+^ load, given by the calculation of ΔF/F integral normalized to control. *p<0.05. (**c**) Representative Western blot showing co-immunoprecipitation of NR2B and NR1 with PP1α using a PP1α antibody in mouse hippocampus lysates. No protein interaction was observed with non-specific IgG. PP1α and IgG immunoprecipitation was performed using a comparable amount of antibodies, and samples were loaded on the same blot (same exposure). (**d**) PP1 directly dephosphorylates Ser1303 *in vitro*. Immunoblot analyses showing the time course of PP1-mediated dephosphorylation on Ser1303 from a GST-fused NR2B C-terminal peptide. GST-NR2B (aa 1221–1482) was incubated with active PP1 following Ser1303 phosphorylation by CaMKII. NR2B was used as loading control.

To confirm the specific effect of NR2B Ser1303 phosphorylation on Ca^2+^ overload, we next tested whether a loss of this residue abolishes such overload. We co-expressed a native (NR2B S1303 WT) or mutated NR2B subunit carrying a Ser1303 to Ala1303 substitution (NR2B S1303A) that prevents phosphorylation, with a highly sensitive Ca^2+^ indicator [28] (TN-XXL) using biolistic transfection in organotypic hippocampal slices. The analysis of isolated post-synaptic NMDAR-dependent Ca^2+^ influx in CA1 pyramidal neurons expressing native NR2B revealed a rapid and transient two-fold increase in somatic [Ca^2+^]_i_ during OGD (**Figure 4a, b**). This increase was prevented by blockade of NR2B Ser1303 phosphorylation in slices expressing NR2B S1303A. The effect of NR2B S1303A expression was not resulting from incorrect trafficking to the surface as observed in transfected Neuro-2a cells (**Figure S3 and Supporting Methods S1**). Moreover, there was no significant difference in TN-XXL expression when comparing cells transfected with the different NR2B variants (**Figure S4 and Supporting Methods S1**). These data overall strongly suggest that Ser1303 phosphorylation is required for OGD-induced Ca^2+^ overload.

Following the demonstration that OGD-induced Ca^2+^ overload and the resulting cellular damage are prevented by PP1α (**Figure 2**), we next hypothesized that PP1α mediates its neuroprotective effect by dephosphorylating Ser1303. To test this hypothesis, we determined whether PP1α associates with NR2B, and whether this correlates with NR2B dephosphorylation. Co-immunoprecipitation assays in mouse hippocampal extracts using anti-PP1α, anti-NR2B or -NR1 antibodies revealed that PP1α does interact with NR2B and NR1 subunits (**Figure 4c**). These results are in agreement with a previous report showing that PP1 forms a complex with the NMDAR [16]. Further, in the presence of a purified NR2B fragment (aa 1221–1482) phosphorylated by CaMKII, PP1α was able to fully dephosphorylate Ser1303 within 30 min *in vitro* (**Figure 4d**). These results provide further evidence for a direct effect of PP1α on NR2B Ser1303 [42].

### NR2B Ser1303 phosphorylation alters NMDAR functions and CREB-dependent gene expression

**Figure 5:**
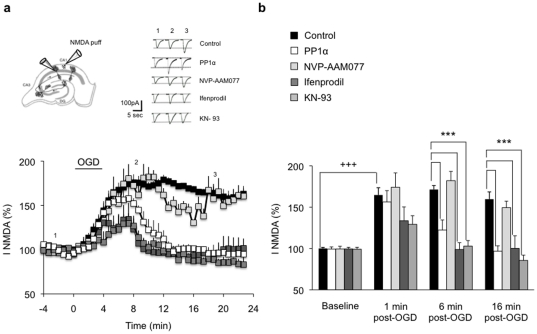
PP1α expression and CaMIIα inhibition normalize NR2B-containing NMDAR-mediated currents after excitotoxicity. (**a**) Significant enhancement of NMDAR currents following 4 min OGD in control slices (control, n = 6). Expression of PP1α (n = 5), ifenprodil treatment (n = 8), and KN-93 treatment (n = 6) led to a full recovery of NMDAR currents 6 min after the end of OGD. NVP-AAM077 (n = 5) has no effect on NMDAR currents that remain potentiated throughout recording. Top left, Schematic representation of an organotypic hippocampal slice showing the positioning of the recording patch-clamp electrode and the puffing pipette. Top right, Individual responses from single CA1 pyramidal neurons before (1), during (2) and 10 min after (3) OGD. (**b**) Time course of OGD mean effect on NMDAR currents. ^+++^p<0.001, ***p<0.001.

NR2B Ser1303 phosphorylation is associated with increased [Ca^2+^]_i_, and Ca^2+^ is known to modulate NMDAR activity. Therefore, we examined whether NMDAR currents are altered by OGD. Isolated post-synaptic NMDAR currents were induced by pressure application of NMDA onto CA1 neurons and recorded in voltage clamp whole-cell configuration. In control slices, NMDAR currents progressively increased during the 4-min OGD and reached a plateau at about 168% of baseline, 2 min after the end of OGD (**Figure 5a, b**). This increase was stable (over 25 min), indicating a persistent potentiation of NMDAR currents by OGD, consistent with previous reports [37]. While NVP-AAM077 had no effect on the potentiation of NMDAR currents, ifenprodil abolished this potentiation and induced a full recovery of NMDAR currents a few minutes after OGD (**Figure 5a, b**). These results confirm that NR2B underlies the prolonged potentiation of NMDAR currents induced by excitotoxicity.

Our previous work has shown that PP1 influences the recovery of fEPSPs from OGD in hippocampus. Its inhibition by specific inhibitors or after induction of long-term potentiation (LTP) prevents fEPSPs recovery while, in contrast, its activation by induction of long-term depression (LTD) restores fEPSPs [21]. Since PP1 contributes to the regulation of NMDARs [19] and blocks excitotoxic [Ca^2+^]_i_ overload, we next examined whether it has a positive effect on fEPSPs recovery and NMDAR currents after OGD. When PP1α was expressed in hippocampal slices, the transient decrease in fEPSPs and increase in NMDAR currents induced by OGD were abolished, and both fEPSPs and NMDAR currents fully recovered (**Figure 5a,b** **, Figure S5 and Supporting Methods S1**). Further, consistent with the implication of CaMKII in [Ca^2+^]_i_ overload, CaMKII inhibition by KN-93 prior to OGD significantly reduced the potentiation of NMDARs currents, and led to a full recovery of these currents (**Figure 5a,b**).

Since protein tyrosine kinases of the Src family are involved in the regulation of NMDAR functions [43] and are associated with excitotoxicity [44], [45], we also investigated Tyr phosphorylation of NR2B. We examined Tyr1472, a residue phosphorylated by Src kinases and associated with NMDAR stabilization at the membrane [46]. Tyr1472 phosphorylation was increased by 1.5 fold (+/−0.3) 1 min after OGD and by 1.9 fold (+/−0.5) after 16 min (**Figure S6a**). This increase was prevented by the tyrosine kinase inhibitor PP2 and by CaMKII inhibition with KN-93, while no effect was observed in non-OGD conditions. These results confirm the involvement of Tyr phosphorylation of NR2B in OGD and suggest a potential link between Tyr and Ser/Thr phosphorylation pathways.

Finally, since excitotoxicity is associated with disruption of cAMP response element-binding protein (CREB)-dependent pathways in CA1 neurons [47], [48], we examined whether CREB phosphorylation on Ser133 and CREB-dependent gene expression are altered by OGD. On Western blots, we observed that CREB phosphorylation on Ser133 was significantly decreased 1 min after OGD (by 0.47 fold+/−0.09) and returned to baseline within 16 min (**Figure S6b**). This decrease was prevented by CaMKII inhibition with KN-93, consistent with the fact that it occurred at the same time point (1 minute after OGD) as CaMKII-mediated NR2B phosphorylation and [Ca^2+^]_i_ overload. Further, it was accompanied by reduced expression of the CREB target genes, pro-survival marker B-cell lymphoma 2 (*Bcl-2*) and the immediate early gene *c-fos* [49], about 20 minutes after OGD (0.86 fold+/−0.02 and 0.76 fold+/−0.07). Importantly, this reduction was fully reversed by PP1α overexpression, confirming the critical role of PP1α in the reversal of the effects of excitotoxicity (**Figure S6c**).

## Discussion

This study identifies a novel mechanism for the control of the switch between cell death and cell survival pathways in hippocampal neurons that involves NR2B-containing NMDARs and the protein phosphatase PP1α. The data demonstrate that the selective phosphorylation of NR2B on Ser1303 by CaMKII is an initial trigger for intraneuronal Ca^2+^ overload during excitotoxicity and that dephosphorylation of this residue by PP1α confers neuroprotection. They further show that the effect of PP1α is mediated by a normalization of NMDAR currents followed by a decrease in Ca^2+^ overload, a restoration of CREB-dependent gene expression, and prevention of delayed neuronal cell death.

These findings are of primary importance for the understanding of the mechanisms of regulation of the NMDAR in adult hippocampal neurons because they newly reveal how the Ser/Thr protein phosphatase PP1α regulates NMDAR functions by acting on a single residue (Ser1303) located on NR2B. Protein phosphorylation is recognized to be a critical regulation mechanism of NMDAR functions, but the residues and enzymes involved in the adult hippocampus have to date only been partially identified. This is particularly true for Ser/Thr phosphorylation, which is known to occur on Ser1303 and 1323 on NR2B and to involve DAPK1, CaMKII, and PKC [14], [15], [17]. However, the reversal of phosphorylation by protein phosphatases remains unclear. The identification of PP1α as a specific phosphatase of Ser1303 on NR2B provides new insight onto the modes of regulation of the NMDAR in hippocampal neurons [20], [50]. It will be of interest in the future to assess the mechanisms by which Ser1303 phosphorylation/dephosphorylation modulates NMDAR functions, this might involve conformational changes [5], a change in NMDAR channel open probability [19], and/or reduced coupling with downstream signaling pathways.

The fact that PP1α co-localizes with NR2B and acts directly on this subunit suggests that it is strategically positioned at the NMDAR to initiate downstream signaling. There, it appears to negatively modulate pathways regulating intracellular Ca^2+^ homeostasis, and Ca^2+^-dependent enzymes such as CaMKIIα. The mechanisms recruited to limit Ca^2+^ entry are not known but may involve L-type voltage-gated Ca^2+^ channels [51], Ca^2+^-permeable AMPA receptors [52], [53], and/or acid-sensing ion channels [54], which may be overactivated by CaMKIIα-mediated phosphorylation. PP1α may also favor neuroprotective signaling cascades involving group I metabotropic glutamate receptors α (mGluR1α), PI3 kinase or Akt, and/or perhaps limits mGluR1α-dependent Ca^2+^ release from intracellular stores that participate to excitotoxic Ca^2+^ overload [55].

Signaling cascades downstream of NMDARs also contribute to pro-apoptotic programs in ischemic conditions. Activation of NR2B-containing NMDARs by CaMKIIα triggers a Ca^2+^ imbalance that dramatically alters cellular properties and leads to delayed cell death. This at the same time also lowers PP1 activity [21]. Consistently, we demonstrate that increased PP1α activity counteracts the Ca^2+^ overload similarly to CaMKIIα inhibition and NR2B-containing NMDAR blockade. Further, the inhibition of PP1 prolongs Ca^2+^ influx, suggesting that endogenous PP1 can restore a normal concentration of intracellular Ca^2+^ upon excitotoxic insult. *In vivo*, the mechanisms leading to PP1 activation or inhibition in the hippocampus are not fully understood but may involve inhibitory targeting partners or inhibitors such as inhibitor-1 [56].

The contribution of NMDARs to excitotoxicity contrasts with the positive role of the receptor in normal excitatory neurotransmission, synaptic plasticity, and memory processes [57]. Such dual functions may be mediated by different populations of NMDARs coupled to distinct interacting partners and/or signaling cascades. NMDAR-dependent pro-death signaling involves NR2B coupling to the stress-activated protein kinase p38 in neurons [58]. Blockade of NR2B and p38 interaction reduces excitotoxic neuronal death without affecting NMDAR-mediated Ca^2+^ influx and synaptic plasticity, suggesting a specific role of NR2B-containing NMDAR downstream signaling in neuronal death.

For some forms of synaptic plasticity such as LTP, NR2B-containing NMDARs associate with CaMKII following the recruitment of the kinase to the receptor [59]. In contrast, synaptic depression such as LTD induces the recruitment of PP1 to NMDARs [20], [21], most likely to extrasynaptic NR2B-containing NMDARs which are negatively regulated during LTD in the hippocampus [60]. Together with these findings, the present data suggest that PP1 may promote LTD by lowering NR2B-containing NMDAR activity, and operate extrasynaptically [61]. Finally, these findings strengthen previous reports showing that signaling through extrasynaptic NMDARs promotes cell death [48], and provide novel perspectives for its blockade by PP1. They underscore the potential of pharmacological approaches targeting protein phosphatases rather than approaches based on NMDAR antagonists, which have severe side effects [62], for the treatment of excitotoxicity in pathologies such as ischemic stroke. These data together with a previous report showing that PP1 inhibition improves memory in aged mice [36] support the therapeutic potential of PP1 modulation in brain disorders and aging.

## Supporting Information

Figure S1 Somato-dendritic distribution of PP1α in hippocampal neurons. (**a**) Cultured hippocampal neurons (DIV11) were transfected with the PP1α-EGFP construct, and fixed 4 days later. Confocal laser microscopy images of a hippocampal pyramidal neuron expressing EGFP-tagged PP1α (green fluorescence, A–C). PP1α is enriched at dendritic spines, as shown at higher magnification (B, C). Scale bar represents 40 µm in A, 20 µm in B, and 10 µm in C. (**b**) PP1α (PP1α-EGFP) and PP1γ (PP1γ-EGFP) subcellular localization. PP1α is excluded from the nucleus, whereas PP1γ is enriched in the nucleus. Scale bar: 30 µm.
(TIF)

Figure S2 PP1 inhibition increases OGD-mediated Ca^2+^ overload. (**a**) I-1 expression in organotypic hippocampal cultures. I-1 transgene mRNA is detected in slices prepared from I-1 transgenic mice and treated with doxycycline (I-1). No I-1 expression in I-1 slices not treated with doxycycline (I-1 no dox) or in doxycycline-treated wild-type slices (WT). –RT (no reverse transcription) and H_2_O as PCR negative controls. (**b**) I-1 expression prolongs Ca^2+^ influx (left panel) and increases overall [Ca^2+^]_i_ load (right panel) upon OGD as seen by ΔF/F ratio (% relative to basal level) and normalized ΔF/F integral in control and I-1 slices (control, n = 7; I-1, n = 5). *p<0.05.
(TIF)

Figure S3 The NR2B S1303 mutant subunit is properly expressed and addressed to the membrane. Cultured Neuro-2a cells were co-transfected with expression vectors for the NR1 subunit and for N-terminally green fluorescent protein-tagged mutated NR2B at Ser1303 (GFP-NR2B S1303A) or GFP-tagged wild-type NR2B (GFP-NR2B WT) subunit. Cells were fixed 4 days later and incubated with GFP and NR2B antibodies without permeabilization. Fluorescent secondary antibodies were applied and cells were imaged with a fluorescent microscope. Surface GFP (green) and NR2B (red) staining was simultaneously observed in NR1/GFP-NR2B S1303A and NR1/GFP-NR2B WT transfected neuro-2a cells. Scale bar: 30 µm.
(TIF)

Figure S4 Co-expression of the Ca^2+^ indicator TN-XXL and a native (NR2B S1303 WT) or a mutated NR2B subunit (NR2B S1303A) upon biolistic transfection in organotypic hippocampal slices. (**a**) Image of a CA1 neuron expressing TN-XXL as shown by green fluorescence. Neuronal layers of the hippocampus are visualized by NeuN staining (red). (**b**) High magnification images of TN-XXL fluorescence in NR2B S1303 WT (top panel) and NR2B S1303A expressing neurons (bottom panel).
(TIF)

Figure S5 Effect of transient OGD on field extracellular post-synaptic potentials (f-EPSP) slope in area CA1 of organotypic hippocampal slices injected with a control virus (control), a PP1α-expressing virus (PP1α) or not injected (non-injected). Quantitative histogram (right panel) of mean f-EPSP slope (over the last 20 min of recording) showing a significant increase in f-EPSP slope recovery in slices overexpressing PP1α (n = 6) compared to control (n = 7) or non-injected slices (n = 9). ***p<0.001. Schematic representation of an organotypic hippocampal slice with the stimulating electrode positioned on Schaffer collateral fibers and the recording electrode within the stratum radiatum (left inset). Individual responses from single slices before (1), during (2) and 10 min after (3) OGD (right inset).
(TIF)

Figure S6 NR2B Ser1303 phosphorylation initiates cell death pathways upon OGD. (**a**) Representative Western blots and corresponding quantitative analysis of NR2B Tyr1472 phosphorylation. Increased level of phospho-NR2B in control slices 1 min after OGD (control 1 min post-OGD, n = 6), and 16 min after OGD (control 16 min post-OGD, n = 5). KN-93 and PP2 treatments block these increases both 1 min post-OGD (KN-93 1 min post-OGD, n = 5; PP2 1 min post-OGD, n = 6) and 16 min post-OGD (KN-93 16 min post-OGD, n = 6; PP2 16 min post-OGD, n = 6). Phospho-protein levels were normalized to non-phosphorylated protein levels and β-actin was used as a loading control. Quantitative data for each condition were normalized to levels of non-OGD condition (control non-OGD, n = 7) from the same blot and exposure. *p<0.05, **p<0.01. (**b**) Representative Western blots and corresponding quantitative analysis of CREB Ser133 phosphorylation. CREB phosphorylation was significantly decreased 1 min after OGD (control 1 min post-OGD, n = 8) with no significant change at 16 min (control 16 min post-OGD, n = 7) in control slices. PP1α expression avoids phospho-CREB depletion 1 min post-OGD. Phospho-protein levels were normalized to non-phosphorylated protein levels and β-actin was used as a loading control. Quantitative data for each condition were normalized to levels of non-OGD condition (control non-OGD, n = 8) from the same blot and exposure. *p<0.05. (**c**) Quantitative RT-PCR data showing a significant reduction in Bcl-2 mRNA level and decreased c-fos mRNA level in control slices subjected to OGD (Bcl-2, n = 9; c-fos, n = 8) compared to non-OGD slices (Bcl-2, n = 12; c-fos, n = 3). PP1α significantly up-regulates Bcl-2 expression (n = 9) and increases c-fos mRNA level (n = 9). Data are expressed as relative quantification. *p<0.05.
(TIF)

Methods S1 Methods for data presented in Supporting Figures: Neuronal culture transfection, I1 RT-PCR, Neuro-2a cell transfection and immunocytochemistry, Field recording.
(DOC)

## References

[1] Arundine M, Tymianski M (2004). Molecular mechanisms of glutamate-dependent neurodegeneration in ischemia and traumatic brain injury.. Cell Mol Life Sci.

[2] Ghosh A, Greenberg ME (1995). Calcium signaling in neurons: molecular mechanisms and cellular consequences.. Science.

[3] Stanika RI, Pivovarova NB, Brantner CA, Watts CA, Winters CA (2009). Coupling diverse routes of calcium entry to mitochondrial dysfunction and glutamate excitotoxicity.. Proc Natl Acad Sci U S A.

[4] Krupp JJ, Vissel B, Heinemann SF, Westbrook GL (1996). Calcium-dependent inactivation of recombinant N-methyl-D-aspartate receptors is NR2 subunit specific.. Mol Pharmacol.

[5] Dingledine R, Borges K, Bowie D, Traynelis SF (1999). The glutamate receptor ion channels.. Pharmacol Rev.

[6] Lein ES, Hawrylycz MJ, Ao N, Ayres M, Bensinger A (2007). Genome-wide atlas of gene expression in the adult mouse brain.. Nature.

[7] Tovar KR, Westbrook GL (1999). The incorporation of NMDA receptors with a distinct subunit composition at nascent hippocampal synapses in vitro.. J Neurosci.

[8] Thomas CG, Miller AJ, Westbrook GL (2006). Synaptic and extrasynaptic NMDA receptor NR2 subunits in cultured hippocampal neurons.. J Neurophysiol.

[9] Zhang SJ, Steijaert MN, Lau D, Schutz G, Delucinge-Vivier C (2007). Decoding NMDA receptor signaling: identification of genomic programs specifying neuronal survival and death.. Neuron.

[10] Fellin T, Pascual O, Gobbo S, Pozzan T, Haydon PG (2004). Neuronal synchrony mediated by astrocytic glutamate through activation of extrasynaptic NMDA receptors.. Neuron.

[11] Jourdain P, Bergersen LH, Bhaukaurally K, Bezzi P, Santello M (2007). Glutamate exocytosis from astrocytes controls synaptic strength.. Nat Neurosci.

[12] Cheng C, Fass DM, Reynolds IJ (1999). Emergence of excitotoxicity in cultured forebrain neurons coincides with larger glutamate-stimulated [Ca(2+)](i) increases and NMDA receptor mRNA levels.. Brain Res.

[13] Cull-Candy S, Brickley S, Farrant M (2001). NMDA receptor subunits: diversity, development and disease.. Curr Opin Neurobiol.

[14] Tu W, Xu X, Peng L, Zhong X, Zhang W DAPK1 interaction with NMDA receptor NR2B subunits mediates brain damage in stroke.. Cell.

[15] Omkumar RV, Kiely MJ, Rosenstein AJ, Min KT, Kennedy MB (1996). Identification of a phosphorylation site for calcium/calmodulindependent protein kinase II in the NR2B subunit of the N-methyl-D-aspartate receptor.. J Biol Chem.

[16] Westphal RS, Tavalin SJ, Lin JW, Alto NM, Fraser ID (1999). Regulation of NMDA receptors by an associated phosphatase-kinase signaling complex.. Science.

[17] Liao GY, Wagner DA, Hsu MH, Leonard JP (2001). Evidence for direct protein kinase-C mediated modulation of N-methyl-D-aspartate receptor current.. Mol Pharmacol.

[18] Wang YT, Salter MW (1994). Regulation of NMDA receptors by tyrosine kinases and phosphatases.. Nature.

[19] Wang LY, Orser BA, Brautigan DL, MacDonald JF (1994). Regulation of NMDA receptors in cultured hippocampal neurons by protein phosphatases 1 and 2A.. Nature.

[20] Morishita W, Connor JH, Xia H, Quinlan EM, Shenolikar S (2001). Regulation of synaptic strength by protein phosphatase 1.. Neuron.

[21] Hedou GF, Koshibu K, Farinelli M, Kilic E, Gee CE (2008). Protein phosphatase 1-dependent bidirectional synaptic plasticity controls ischemic recovery in the adult brain.. J Neurosci.

[22] Newell DW, Barth A, Papermaster V, Malouf AT (1995). Glutamate and non-glutamate receptor mediated toxicity caused by oxygen and glucose deprivation in organotypic hippocampal cultures.. J Neurosci.

[23] Szulc J, Wiznerowicz M, Sauvain MO, Trono D, Aebischer P (2006). A versatile tool for conditional gene expression and knockdown.. Nat Methods.

[24] Ceulemans H, Vulsteke V, De Maeyer M, Tatchell K, Stalmans W (2002). Binding of the concave surface of the Sds22 superhelix to the alpha 4/alpha 5/alpha 6-triangle of protein phosphatase-1.. J Biol Chem.

[25] Gahwiler BH, Capogna M, Debanne D, McKinney RA, Thompson SM (1997). Organotypic slice cultures: a technique has come of age.. Trends Neurosci.

[26] Livak KJ, Schmittgen TD (2001). Analysis of relative gene expression data using real-time quantitative PCR and the 2(-Delta Delta C(T)) Method.. Methods.

[27] Helmchen F, Imoto K, Sakmann B (1996). Ca2+ buffering and action potential-evoked Ca2+ signaling in dendrites of pyramidal neurons.. Biophys J.

[28] Mank M, Santos AF, Direnberger S, Mrsic-Flogel TD, Hofer SB (2008). A genetically encoded calcium indicator for chronic in vivo two-photon imaging.. Nat Methods.

[29] Chung HJ, Huang YH, Lau LF, Huganir RL (2004). Regulation of the NMDA receptor complex and trafficking by activity-dependent phosphorylation of the NR2B subunit PDZ ligand.. J Neurosci.

[30] Strasser U, Fischer G (1995). Quantitative measurement of neuronal degeneration in organotypic hippocampal cultures after combined oxygen/glucose deprivation.. J Neurosci Methods.

[31] Lipton P (1999). Ischemic cell death in brain neurons.. Physiol Rev.

[32] Schubert P, Keller F, Nakamura Y, Rudolphi K (1994). The use of ion-sensitive electrodes and fluorescence imaging in hippocampal slices for studying pathological changes of intracellular Ca2+ regulation.. J Neural Transm.

[33] Williams K (1993). Ifenprodil discriminates subtypes of the N-methyl-D-aspartate receptor: selectivity and mechanisms at recombinant heteromeric receptors.. Mol Pharmacol.

[34] Auberson YP, Allgeier H, Bischoff S, Lingenhoehl K, Moretti R (2002). 5-Phosphonomethylquinoxalinediones as competitive NMDA receptor antagonists with a preference for the human 1A/2A, rather than 1A/2B receptor composition.. Bioorg Med Chem Lett.

[35] Bartlett TE, Bannister NJ, Collett VJ, Dargan SL, Massey PV (2007). Differential roles of NR2A and NR2B-containing NMDA receptors in LTP and LTD in the CA1 region of two-week old rat hippocampus.. Neuropharmacology.

[36] Genoux D, Haditsch U, Knobloch M, Michalon A, Storm D (2002). Protein phosphatase 1 is a molecular constraint on learning and memory.. Nature.

[37] Gee CE, Benquet P, Raineteau O, Rietschin L, Kirbach SW (2006). NMDA receptors and the differential ischemic vulnerability of hippocampal neurons.. Eur J Neurosci.

[38] Miller SG, Patton BL, Kennedy MB (1988). Sequences of autophosphorylation sites in neuronal type II CaM kinase that control Ca2(+)-independent activity.. Neuron.

[39] Schworer H, Kilbinger H (1988). Enhancement of guinea-pig intestinal peristalsis by blockade of muscarinic M1-receptors.. Br J Pharmacol.

[40] Thiel G, Czernik AJ, Gorelick F, Nairn AC, Greengard P (1988). Ca2+/calmodulin-dependent protein kinase II: identification of threonine-286 as the autophosphorylation site in the alpha subunit associated with the generation of Ca2+-independent activity.. Proc Natl Acad Sci U S A.

[41] Strack S, Barban MA, Wadzinski BE, Colbran RJ (1997). Differential inactivation of postsynaptic density-associated and soluble Ca2+/calmodulin-dependent protein kinase II by protein phosphatases 1 and 2A.. J Neurochem.

[42] Raveendran R, Devi Suma Priya S, Mayadevi M, Steephan M, Santhoshkumar TR (2009). Phosphorylation status of the NR2B subunit of NMDA receptor regulates its interaction with calcium/calmodulin-dependent protein kinase II.. J Neurochem.

[43] Salter MW, Kalia LV (2004). Src kinases: a hub for NMDA receptor regulation.. Nat Rev Neurosci.

[44] Camacho A, Montiel T, Massieu L (2007). Sustained metabolic inhibition induces an increase in the content and phosphorylation of the NR2B subunit of N-methyl-D-aspartate receptors and a decrease in glutamate transport in the rat hippocampus in vivo.. Neuroscience.

[45] Lennmyr F, Ericsson A, Gerwins P, Akterin S, Ahlstrom H (2004). Src family kinase-inhibitor PP2 reduces focal ischemic brain injury.. Acta Neurol Scand.

[46] Prybylowski K, Chang K, Sans N, Kan L, Vicini S (2005). The synaptic localization of NR2B-containing NMDA receptors is controlled by interactions with PDZ proteins and AP-2.. Neuron.

[47] Walton M, Sirimanne E, Williams C, Gluckman P, Dragunow M (1996). The role of the cyclic AMP-responsive element binding protein (CREB) in hypoxic-ischemic brain damage and repair.. Brain Res Mol Brain Res.

[48] Hardingham GE, Fukunaga Y, Bading H (2002). Extrasynaptic NMDARs oppose synaptic NMDARs by triggering CREB shut-off and cell death pathways.. Nat Neurosci.

[49] Kitagawa K (2007). CREB and cAMP response element-mediated gene expression in the ischemic brain.. Febs J.

[50] Lieberman DN, Mody I (1994). Regulation of NMDA channel function by endogenous Ca(2+)-dependent phosphatase.. Nature.

[51] Fields RD, Lee PR, Cohen JE (2005). Temporal integration of intracellular Ca2+ signaling networks in regulating gene expression by action potentials.. Cell Calcium.

[52] Liu S, Lau L, Wei J, Zhu D, Zou S (2004). Expression of Ca(2+)-permeable AMPA receptor channels primes cell death in transient forebrain ischemia.. Neuron.

[53] Barria A, Muller D, Derkach V, Griffith LC, Soderling TR (1997). Regulatory phosphorylation of AMPA-type glutamate receptors by CaM-KII during long-term potentiation.. Science.

[54] Gao J, Duan B, Wang DG, Deng XH, Zhang GY (2005). Coupling between NMDA receptor and acid-sensing ion channel contributes to ischemic neuronal death.. Neuron.

[55] Xu W, Wong TP, Chery N, Gaertner T, Wang YT (2007). Calpain-mediated mGluR1alpha truncation: a key step in excitotoxicity.. Neuron.

[56] Blitzer RD, Connor JH, Brown GP, Wong T, Shenolikar S (1998). Gating of CaMKII by cAMP-regulated protein phosphatase activity during LTP.. Science.

[57] Lee YS, Silva AJ (2009). The molecular and cellular biology of enhanced cognition.. Nat Rev Neurosci.

[58] Soriano FX, Martel MA, Papadia S, Vaslin A, Baxter P (2008). Specific targeting of pro-death NMDA receptor signals with differing reliance on the NR2B PDZ ligand.. J Neurosci.

[59] Barria A, Malinow R (2005). NMDA receptor subunit composition controls synaptic plasticity by regulating binding to CaMKII.. Neuron.

[60] Kollen M, Dutar P, Jouvenceau A (2008). The magnitude of hippocampal long term depression depends on the synaptic location of activated NR2-containing N-methyl-D-aspartate receptors.. Neuroscience.

[61] Martel MA, Wyllie DJ, Hardingham GE (2008). In developing hippocampal neurons, NR2B-containing N-methyl-d-aspartate receptors (NMDARs) can mediate signaling to neuronal survival and synaptic potentiation, as well as neuronal death.. Neuroscience.

[62] Ikonomidou C, Turski L (2002). Why did NMDA receptor antagonists fail clinical trials for stroke and traumatic brain injury?. Lancet Neurol.

