# Supplementary figures and images for: Selective Regulation of NR2B by Protein Phosphatase-1 for the Control of the NMDA Receptor in Neuroprotection
Source: PLoS One. 2012 Mar 30;7(3):e34047. doi: 10.1371/journal.pone.0034047 (PMC3316588; doi:10.1371/journal.pone.0034047)

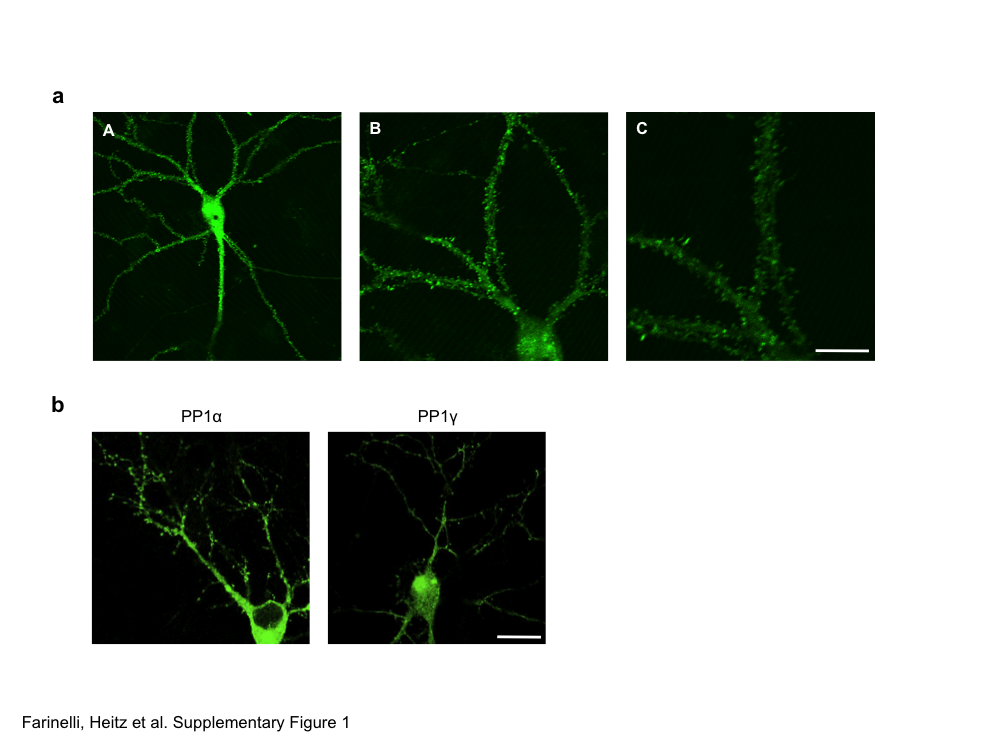

Supplement: Figure S1 — Somato-dendritic distribution of PP1α in hippocampal neurons. (a) Cultured hippocampal neurons (DIV11) were transfected with the PP1α-EGFP construct, and fixed 4 days later. Confocal laser microscopy images of a hippocampal pyramidal neuron expressing EGFP-tagged PP1α (green fluorescence, A–C). PP1α is enriched at dendritic spines, as shown at higher magnification (B, C). Scale bar represents 40 µm in A, 20 µm in B, and 10 µm in C. (b) PP1α (PP1α-EGFP) and PP1γ (PP1γ-EGFP) subcellular localization. PP1α is excluded from the nucleus, whereas PP1γ is enriched in the nucleus. Scale bar: 30 µm. (TIF) [file pone.0034047.s001.tif]

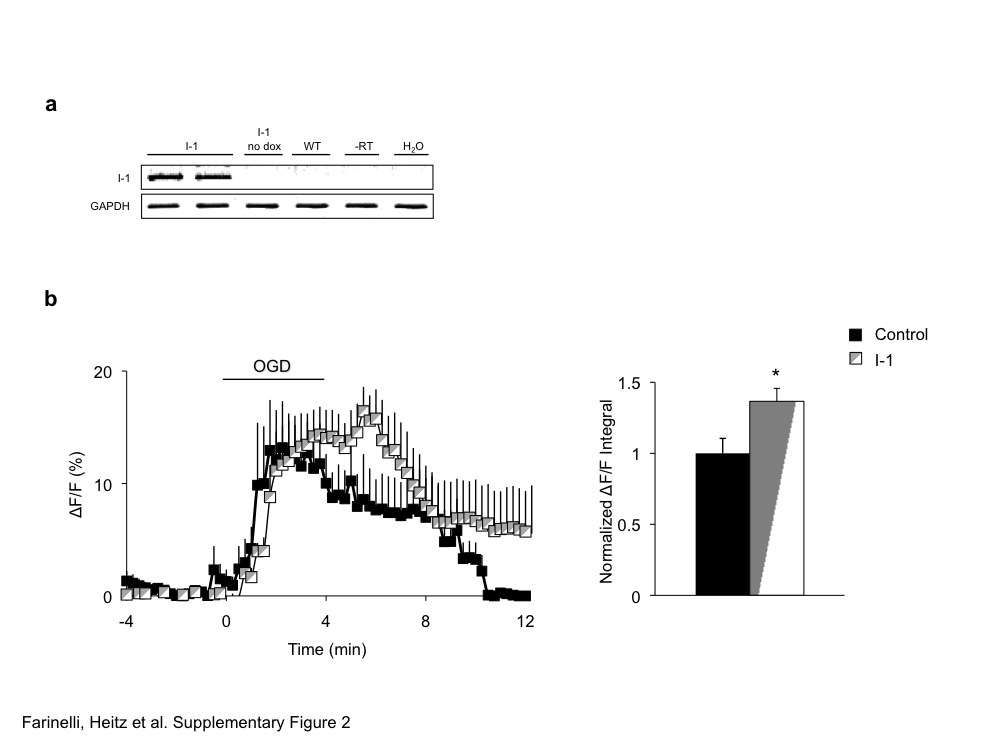

Supplement: Figure S2 — PP1 inhibition increases OGD-mediated Ca2+ overload. (a) I-1 expression in organotypic hippocampal cultures. I-1 transgene mRNA is detected in slices prepared from I-1 transgenic mice and treated with doxycycline (I-1). No I-1 expression in I-1 slices not treated with doxycycline (I-1 no dox) or in doxycycline-treated wild-type slices (WT). –RT (no reverse transcription) and H2O as PCR negative controls. (b) I-1 expression prolongs Ca2+ influx (left panel) and increases overall [Ca2+]i load (right panel) upon OGD as seen by ΔF/F ratio (% relative to basal level) and normalized ΔF/F integral in control and I-1 slices (control, n = 7; I-1, n = 5). *p<0.05. (TIF) [file pone.0034047.s002.tif]

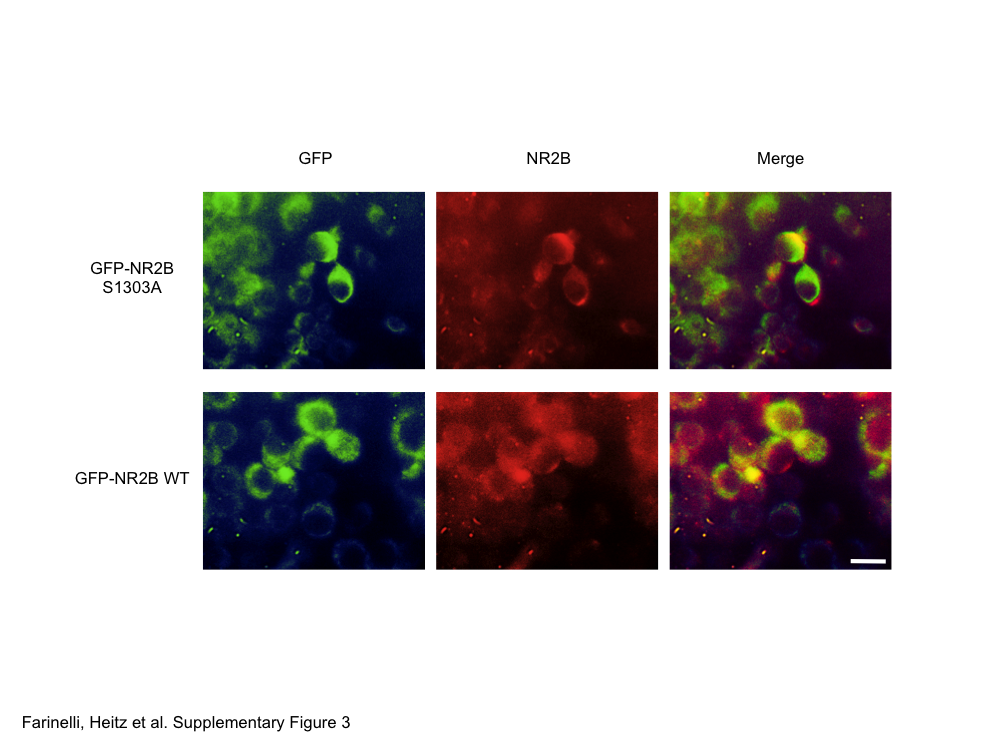

Supplement: Figure S3 — The NR2B S1303 mutant subunit is properly expressed and addressed to the membrane. Cultured Neuro-2a cells were co-transfected with expression vectors for the NR1 subunit and for N-terminally green fluorescent protein-tagged mutated NR2B at Ser1303 (GFP-NR2B S1303A) or GFP-tagged wild-type NR2B (GFP-NR2B WT) subunit. Cells were fixed 4 days later and incubated with GFP and NR2B antibodies without permeabilization. Fluorescent secondary antibodies were applied and cells were imaged with a fluorescent microscope. Surface GFP (green) and NR2B (red) staining was simultaneously observed in NR1/GFP-NR2B S1303A and NR1/GFP-NR2B WT transfected neuro-2a cells. Scale bar: 30 µm. (TIF) [file pone.0034047.s003.tif]

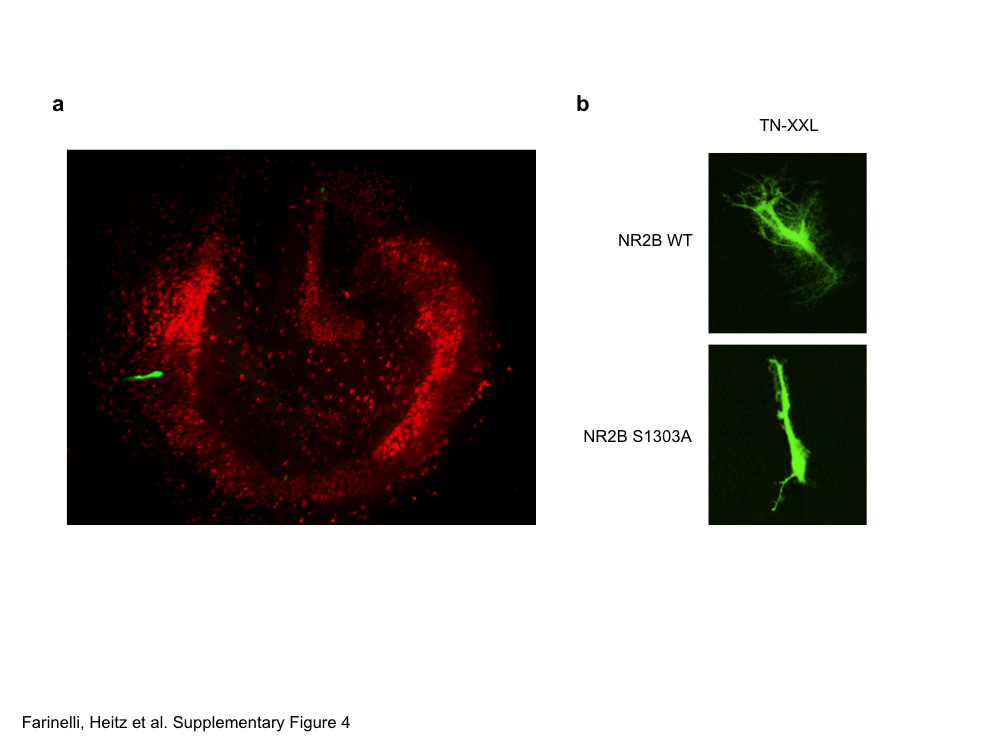

Supplement: Figure S4 — Co-expression of the Ca2+ indicator TN-XXL and a native (NR2B S1303 WT) or a mutated NR2B subunit (NR2B S1303A) upon biolistic transfection in organotypic hippocampal slices. (a) Image of a CA1 neuron expressing TN-XXL as shown by green fluorescence. Neuronal layers of the hippocampus are visualized by NeuN staining (red). (b) High magnification images of TN-XXL fluorescence in NR2B S1303 WT (top panel) and NR2B S1303A expressing neurons (bottom panel). (TIF) [file pone.0034047.s004.tif]

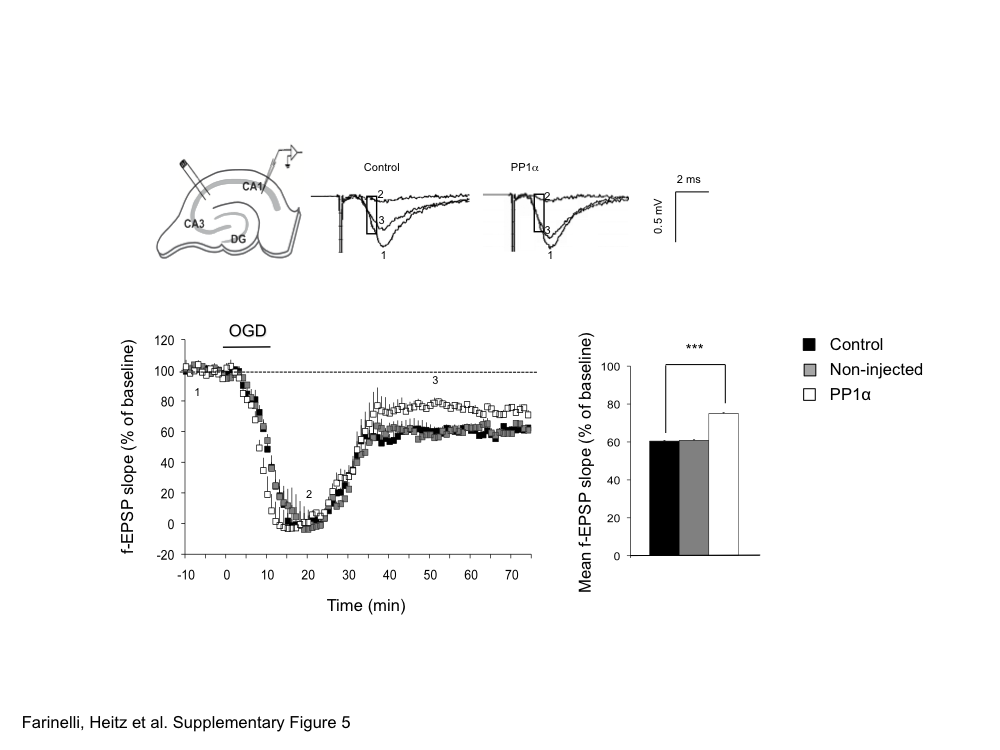

Supplement: Figure S5 — Effect of transient OGD on field extracellular post-synaptic potentials (f-EPSP) slope in area CA1 of organotypic hippocampal slices injected with a control virus (control), a PP1α-expressing virus (PP1α) or not injected (non-injected). Quantitative histogram (right panel) of mean f-EPSP slope (over the last 20 min of recording) showing a significant increase in f-EPSP slope recovery in slices overexpressing PP1α (n = 6) compared to control (n = 7) or non-injected slices (n = 9). ***p<0.001. Schematic representation of an organotypic hippocampal slice with the stimulating electrode positioned on Schaffer collateral fibers and the recording electrode within the stratum radiatum (left inset). Individual responses from single slices before (1), during (2) and 10 min after (3) OGD (right inset). (TIF) [file pone.0034047.s005.tif]

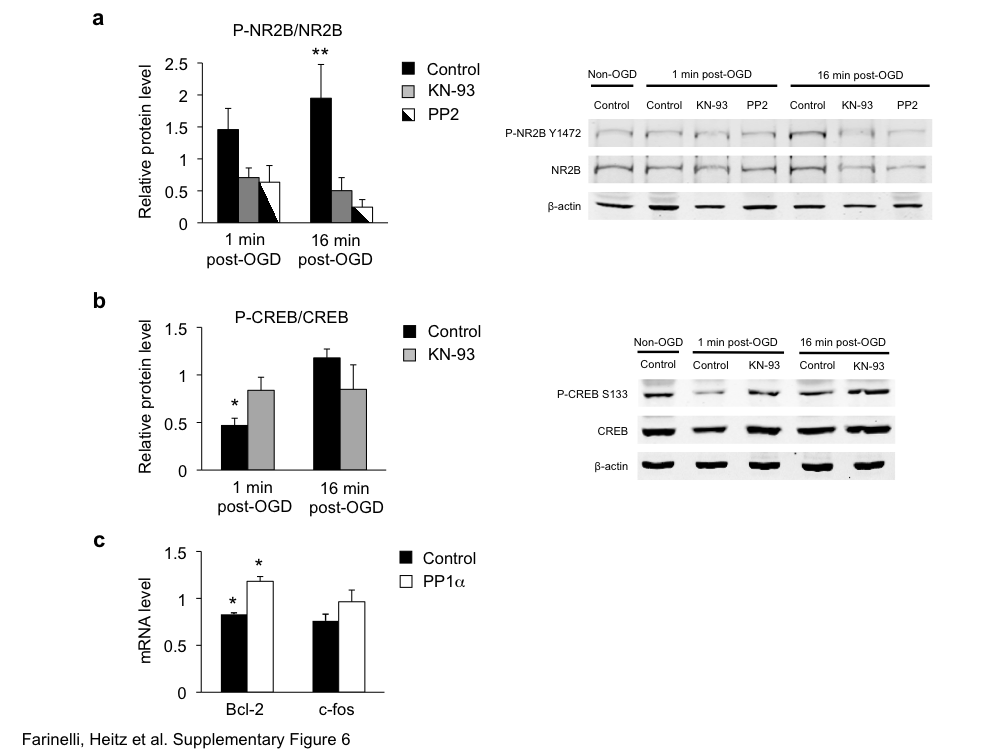

Supplement: Figure S6 — NR2B Ser1303 phosphorylation initiates cell death pathways upon OGD. (a) Representative Western blots and corresponding quantitative analysis of NR2B Tyr1472 phosphorylation. Increased level of phospho-NR2B in control slices 1 min after OGD (control 1 min post-OGD, n = 6), and 16 min after OGD (control 16 min post-OGD, n = 5). KN-93 and PP2 treatments block these increases both 1 min post-OGD (KN-93 1 min post-OGD, n = 5; PP2 1 min post-OGD, n = 6) and 16 min post-OGD (KN-93 16 min post-OGD, n = 6; PP2 16 min post-OGD, n = 6). Phospho-protein levels were normalized to non-phosphorylated protein levels and β-actin was used as a loading control. Quantitative data for each condition were normalized to levels of non-OGD condition (control non-OGD, n = 7) from the same blot and exposure. *p<0.05, **p<0.01. (b) Representative Western blots and corresponding quantitative analysis of CREB Ser133 phosphorylation. CREB phosphorylation was significantly decreased 1 min after OGD (control 1 min post-OGD, n = 8) with no significant change at 16 min (control 16 min post-OGD, n = 7) in control slices. PP1α expression avoids phospho-CREB depletion 1 min post-OGD. Phospho-protein levels were normalized to non-phosphorylated protein levels and β-actin was used as a loading control. Quantitative data for each condition were normalized to levels of non-OGD condition (control non-OGD, n = 8) from the same blot and exposure. *p<0.05. (c) Quantitative RT-PCR data showing a significant reduction in Bcl-2 mRNA level and decreased c-fos mRNA level in control slices subjected to OGD (Bcl-2, n = 9; c-fos, n = 8) compared to non-OGD slices (Bcl-2, n = 12; c-fos, n = 3). PP1α significantly up-regulates Bcl-2 expression (n = 9) and increases c-fos mRNA level (n = 9). Data are expressed as relative quantification. *p<0.05. (TIF) [file pone.0034047.s006.tif]
