# Supplementary material for: Selective Regulation of NR2B by Protein Phosphatase-1 for the Control of the NMDA Receptor in Neuroprotection
Source: PLoS One. 2012 Mar 30;7(3):e34047. doi: 10.1371/journal.pone.0034047 (PMC3316588; doi:10.1371/journal.pone.0034047)
Supplement: Methods S1 — Methods for data presented in Supporting Figures: Neuronal culture transfection, I1 RT-PCR, Neuro-2a cell transfection and immunocytochemistry, Field recording. (DOC) [file pone.0034047.s007.doc]

**SUPPORTING INFORMATION**

**Supporting Methods**

**Neuronal culture transfection**

Rat hippocampal neurons (DIV11) were transfected with pEGFP-C1-PP1α plasmid (500ng) or with pEGFP-C1-PP1α (1μg) and pEGFP-C1-PP1γ (1μg) [1], according to previously described [2]. Cells were fixed in 4% PFA 4 days post-transfection and mounted on glass slides. Images were taken using a Zeiss LSM 410 confocal laser-scanning microscope.

**I-1 RT-PCR**

RNA extraction and reverse transcription was performed as previously mentioned (see quantitative real-time PCR). We used I-1 specific forward (5’-GCTCGAGCCACATCTAGACC-3’) and reverse (5’-CCTCATCCACCTCAGGAGAG-3’) primers. GAPDH was used as an internal control (forward primer: 5’-CACTGAGCATCTCCCTCACA-3’; reverse primer: 5’-GTGGGTGCAGCGAACTTTAT-3’).

**Neuro-2a cell transfection and immunocytochemistry**

Neuro-2a cells were cultured as previously described [3]. The NR1 and GFP-NR2B (wild-type) constructs used for transfection were generously provided by P.Scheiffele (Biozentrum, Basel University, Switzerland) and the GFP-NR2B S1303 mutant was generated by PCR-based mutagenesis (QuikChange Site-Directed Mutagenesis Kit, Stratagene). NR1 and GFP-NR2B or GFP-NR2B S1303 constructs were transiently co-transfected in neuro-2a cells using Lipofectamine 2000 (Invitrogen) and according to manufacturer’s instructions. Four days post-transfection, cells were fixed for 2 h at 4˚C in 4% paraformaldehyde (Sigma), 0.1M phosphate buffer (PB), pH7.4. Cells were washed in 0.1 m PB and blocked in 0.1 M PB, 10% heat-inactivated horse serum (HS; Sigma) for 1 h at 4 °C, and incubated with primary rabbit anti-EGFP (Synaptic Systems) and mouse anti-NR2B (Chemicon) antibodies (1:500) overnight at 4 °C in 0.1 M PB, 2% HS. Cells were then washed in 0.1 M PB and incubated 2 h at 4 °C with goat anti-rabbit FITC and donkey anti-mouse TRITC fluorescence-conjugated secondary antibodies (1:1000; Jackson ImmunoResearch). After washing in 0.1 M PB, cells were mounted on slides. Fluorescent images were acquired with a CoolSNAPK4 digital camera (Roper Scientific) mounted on an Axiophot microscope (Zeiss) and analysed using MCID Elite 7.0 software (MCID).

**Field recording**

Hippocampal slices injected with aCSF, PP1α, PP1α-EGFP or control virus treated with doxycycline were subjected to OGD with simultaneous extracellular recording. Slices were placed in an interface chamber at room temperature and perfused with aCSF (1-2 ml/min). A monopolar electrode was placed in Schaffer collateral fibers, and stimulation pulses were applied at 0.033 Hz (20-80 μA) to evoke field excitatory post-synaptic potentials (fEPSPs) of 0.2-0.5 mV recorded in the stratum radiatum with a borosilicate micropipette filled with aCSF. fEPSPs slope was measured every 30 sec and the OGD effect was expressed relative to pre-conditioning baseline (mean slope over 15 min prior to OGD normalized to 100%). After 15 min baseline, slices were exposed to 10 min OGD by perfusing aCSF deprived of oxygen (saturated with 95% N_2_, 5% CO_2_) and glucose (replaced with 11 mM sucrose). After 10 min, perfusion with normal aCSF was resumed. Recordings were made using an Axopatch 200A amplifier (Axon Instruments), monitored on-line and analyzed off-line using pCLAMP. Data were pooled across slices and expressed as mean ± SEM.

**Supporting References**

1. Ceulemans H, Vulsteke V, De Maeyer M, Tatchell K, Stalmans W, et al. (2002) Binding of the concave surface of the Sds22 superhelix to the alpha 4/alpha 5/alpha 6-triangle of protein phosphatase-1. J Biol Chem 277: 47331-47337.

2. Buerli T, Pellegrino C, Baer K, Lardi-Studler B, Chudotvorova I, et al. (2007) Efficient transfection of DNA or shRNA vectors into neurons using magnetofection. Nat Protoc 2: 3090-3101.

3. Kaneko K, Zulianello L, Scott M, Cooper CM, Wallace AC, et al. (1997) Evidence for protein X binding to a discontinuous epitope on the cellular prion protein during scrapie prion propagation. Proc Natl Acad Sci U S A 94: 10069-10074.
